# Nurses’ Career Progression and Career Success: A Scoping Review of Conceptualisation and Associated Workforce Conditions

**DOI:** 10.1155/jonm/8907139

**Published:** 2026-09-30

**Authors:** Elizabeth Bukola Ogunsemo, Jude Ominyi, Noreen Cushen-Brewster

**Affiliations:** ^1^ School of Nursing Midwifery & Health Education University of Bedfordshire Luton UK beds.ac.uk; ^2^ School of Health Sciences & Society University of Suffolk Ipswich UK uos.ac.uk

**Keywords:** career advancement, career progression, career success, leadership, nursing management, scoping review, workforce

## Abstract

**Background:**

Conceptual inconsistency exists in nursing literature, with career progression, career advancement, career success, professional development and career fulfilment defined and applied in varying ways. This ambiguity blurs important distinctions and limits the comparison and interpretation of findings. This scoping review mapped how career progression and career success have been conceptualised and measured and synthesised the organisational and workforce factors reported in relation to these outcomes.

**Methods:**

We conducted a scoping review using the Arksey and O′Malley framework. PubMed/MEDLINE, CINAHL Complete, PsycINFO, Scopus and Web of Science were searched. Data were charted and synthesised using a descriptive thematic approach. Reporting followed the PRISMA Extension for Scoping Reviews.

**Results:**

Of 2504 records identified, 20 studies were included. Studies included qualitative, quantitative and mixed‐method designs. Career progression was represented by movement through grades, roles or responsibilities, whereas objective career success concerned observable achievements and subjective career success concerned personal evaluations of achievement, recognition or fulfilment. Professional development, identity, belonging and intention to remain were related constructs rather than interchangeable outcomes. Reported factors were organised into five descriptive themes: organisational career infrastructure; leadership and managerial conditions; workforce environment; social and relational conditions; and individual resources and career behaviours.

**Conclusion:**

The literature reports relationships between career‐related outcomes and conditions at individual, relational, organisational and workforce levels, but it does not establish causal or hierarchical effects. Conceptual inconsistency, contextual heterogeneity and the absence of prospective longitudinal and intervention evidence restrict causal interpretation and transferability.

**Implications for Nursing Management:**

Findings support consideration of transparent career pathways, equitable access to development, recognition of prior expertise, supportive career conversations and adequate practice environments. These approaches may be considered within local workforce systems but require prospective evaluation because direct evidence that they improve career progression, retention or workforce sustainability remains limited.

**Summary**
• What is currently known
◦ Nursing career research encompasses promotion, professional development, career satisfaction, fulfilment, identity and retention, often without clearly distinguishing between these constructs.
◦ Organisational support, leadership and practice environments are frequently reported in relation to nurses’ career experiences.
◦ The existing literature is methodologically and contextually heterogeneous.
• What this paper adds
◦ This review distinguishes career progression from objective and subjective career success and identifies professional development, identity and retention as related but non‐equivalent constructs.
◦ The synthesis maps organisational, leadership, workforce, relational and individual conditions reported in relation to career‐related outcomes.
◦ The findings highlight priorities for nursing management consideration and prospective evaluation, while recognising the absence of intervention evidence demonstrating effects on career progression, retention or workforce sustainability.

## 1. Introduction

Healthcare systems continue to face difficulties in sustaining a stable nursing workforce, with concerns extending beyond recruitment to nurses’ opportunities for professional development, progression into expanded roles and the maintenance of meaningful careers [1, 2]. Career opportunities are relevant to nursing management because they intersect with professional recognition, workforce participation, leadership development, engagement and retention [3–5]. However, the underlying literature uses several career terms inconsistently, which can obscure both what has been studied and what the findings can support.

For the purposes of this review, career progression was conceptualised as movement or development over time within or across nursing roles. It may be indicated by promotion, movement through clinical or managerial grades, increased responsibility, an expanded scope of practice or transition into another recognised role. Career advancement was treated more specifically as upward or expanded movement within this broader process, including movement between defined levels of practice or into advanced roles [6]. Career success encompasses positive career outcomes and personal evaluations, comprising both objective and subjective dimensions [7]. Objective career success relates to externally observable outcomes, such as promotion, grade, remuneration, status and leadership responsibility, whereas subjective career success reflects an individual’s evaluation of their career, including satisfaction, perceived achievement, recognition, influence, meaningful work and fulfilment [8].

Professional development refers to the lifelong process through which nurses maintain and extend their knowledge, competence and practice capability, including preparation for new or expanded roles and the pursuit of career goals [3]. It can support career planning and create readiness for progression, particularly where organisations provide structured pathways and appropriate resources; however, participation in professional development does not itself demonstrate movement to a higher grade, role or level of responsibility [4]. Professional identity concerns how nurses understand themselves as nurses and position themselves in relation to professional values, roles, colleagues and the practice context, whereas professional belonging reflects a sense of membership and connection within the nursing community and workplace [9, 10]. Career satisfaction and fulfilment are personal evaluations that may contribute to subjective career success [7, 8]. Intention to remain is a distinct workforce outcome, although nursing research indicates that it may be associated with organisational support and perceived career success [11]. These constructs were therefore treated as related but non‐equivalent. Throughout this review, the term ‘career‐related outcomes’ is used only when the evidence spans more than one of these constructs.

These distinctions are necessary because career research does not employ a single, conceptually uniform outcome. Observable career attainment, personal evaluations of success, professional development, professional identity and workforce retention may be related, but they occupy different positions as outcomes, antecedents, mediators or consequences [3, 7, 8]. Within nursing, this variation is reflected in the use of promotion and role transition as indicators of progression, general and nursing‐specific instruments to measure career success and separate constructs concerning professional identity, belonging and intention to remain [9–12]. Without clear conceptual boundaries, synthesising these constructs together risks conflating distinct processes and outcomes, thereby overstating the coherence of a heterogeneous evidence base.

Existing studies have reported associations between career‐related outcomes and structural empowerment, perceived organisational support, leadership, workplace conditions, professional relationships, self‐efficacy and career‐related behaviours [11, 13–16]. Research involving migrant and minoritised nurses has additionally identified delayed progression, deskilling, underrecognition of prior experience and unequal access to professional development and advancement opportunities [17–19]. However, this evidence is dispersed across nursing management, organisational behaviour, professional development and workforce research. The review aimed to map how career progression and career success have been conceptualised and measured, explore the organisational and workforce factors reported in relation to these career‐related outcomes and identify conceptual, methodological and contextual gaps in the evidence. This review was guided by the following questions:

1. How have career progression and career success been defined and operationalised in nursing research?.
2. What organisational, leadership, workforce and professional factors have been reported in relation to these career‐related outcomes?.
3. What conceptual, methodological and contextual gaps remain within the evidence?.

## 2. Methods

### 2.1. Design, Protocol and Reporting

A scoping review design was selected because this approach is suited to clarifying concepts, mapping the range and characteristics of available evidence and identifying knowledge gaps [20]. Consistent with these purposes, the review was not designed to rank determinants, evaluate intervention effectiveness, pool effect estimates or establish causal relationships. The review followed the five‐stage scoping review framework proposed by Arksey and O′Malley [21], subsequent methodological refinements [22] and guidance in the JBI Manual for Evidence Synthesis [20]. The stages were formulation of the review questions, identification of relevant studies, selection of eligible studies, charting of data and collation and reporting. Reporting followed the PRISMA extension for Scoping Reviews (PRISMA‐ScR) [23]. The completed PRISMA‐ScR checklist is provided as a supporting file.

An apriori protocol specified the review questions, eligibility criteria, information sources, screening procedures and data‐charting fields. The protocol was not registered or published. Research ethics approval was not required because the review analysed published literature. A formal critical appraisal was not undertaken because the purpose was to map, rather than determine the certainty of, the evidence. Nevertheless, study design features and principal methodological limitations were charted and summarised to support appropriately cautious interpretation.

### 2.2. Eligibility Criteria

Eligibility was structured using the Population, Concept and Context framework (Table 1). Empirical qualitative, quantitative and mixed‐methods studies were eligible when they included registered, clinical or staff nurses working in healthcare settings and examined career progression, career advancement, objective career success or subjective career success. Career advancement was treated as a specific upward or expanded form of career progression. Studies examining professional development, professional identity, belonging or retention were eligible only when they reported an explicit relationship with a defined career progression or career‐success outcome.

**TABLE 1:** Population, Concept and Context framework and eligibility.

Element	Operational definition	Eligibility in this review
Population	Registered, clinical or staff nurses working in healthcare settings.	Nurse‐only findings, or nurse findings reported separately from mixed professional groups.
Concept	Career progression or advancement; objective career success; subjective career success.	Related constructs were eligible only when explicitly linked to a defined career progression or success outcome.
Context	Healthcare delivery and employing organisations in any country or setting.	No geographical restriction; full peer‐reviewed English‐language journal articles.

Studies were required to be published as full, peer‐reviewed journal articles in English. No geographical or publication date restrictions were applied. Studies were excluded if they focused exclusively on students, unregistered trainees or non‐nursing occupations; reported only education or training outcomes without an explicit career‐related outcome; or were reviews, editorials, commentaries, protocols, conference abstracts, dissertations, book chapters or abstract‐only records. Studies involving mixed professional groups were eligible only when findings for nurses were reported separately or nurses comprised the defined study population. No date restriction was applied because earlier research could have contributed to the development or operationalisation of career‐related constructs.

### 2.3. Information Sources and Search Strategy

Searches were conducted from database inception in PubMed/MEDLINE, CINAHL Complete, PsycINFO, Scopus and Web of Science. The original searches were completed on 15 January 2026. All five database searches were rerun on 31 March 2026, before final synthesis and writing; 31 March 2026, was the final search date. Database‐specific controlled vocabulary and free‐text terms represented three concepts: nurses, career progression or career success, and organisational, leadership or workforce conditions. Boolean operators, phrase searching, truncation and database indexing were adapted to each source. Tables 2 and 3 present the search framework and full search strategy, providing the complete reproducible strategies, applied limits and final database counts.

**TABLE 2:** Search strategy framework.

Concept	Illustrative terms
Nursing population	nurse∗; registered nurse∗; clinical nurse∗; staff nurse∗; nursing workforce
Career outcome	career success; career progression; career advancement; career development; professional success; professional growth; promotion
Conditions	leadership; management; organisational support; work environment; empowerment; career opportunity; professional development; workforce; mentoring

^∗^Truncation and was used to retrieve variations of a word stem.

**TABLE 3:** Search strategies and search outcomes.

Database	Search strategy	Limits/database scope	Original search date	Updated/final search date	Final hits
PubMed/MEDLINE	(“Nurses”[MeSH] OR nurse∗ OR “registered nurse” OR “clinical nurse” OR “staff nurse∗” OR “nursing workforce”) AND (“career success” OR “career progression” OR “career advancement” OR “career development” OR “professional success” OR “professional growth”) AND (“leadership” OR “management” OR “organizational support” OR “work environment” OR “empowerment” OR “career opportunity” OR “professional development” OR “workforce”	No publication‐date restriction. English‐language, peer‐reviewed full‐article criteria applied during eligibility assessment.	15 January 2026	31 March 2026	528
CINAHL Complete	(MH “Nurses+” OR nurse∗ OR “registered nurse” OR “clinical nurse” OR “staff nurse∗“) AND (“career success” OR “career progression” OR “career advancement” OR “career development” OR “professional growth”) AND (“leadership” OR “management” OR “organizational support” OR “work environment” OR “empowerment” OR “professional development”)	Peer‐reviewed journals; no publication‐date restriction. English‐language eligibility applied during screening.	15 January 2026	31 March 2026	741
PsycINFO	(DE “Nurses” OR nurse∗ OR “registered nurse∗“) AND (“career success” OR “career progression” OR “career advancement” OR “professional growth”) AND (“leadership” OR “organisational support” OR “work environment” OR workforce OR empowerment)	Peer‐reviewed publications; no publication‐date restriction. English‐language eligibility applied during screening.	15 January 2026	31 March 2026	279
Scopus	TITLE‐ABS‐KEY (nurse∗ OR “registered nurse” OR “clinical nurse” OR “staff nurse∗”) AND TITLE‐ABS‐KEY (“career success” OR “career progression” OR “career advancement” OR “career development”) AND TITLE‐ABS‐KEY (leadership OR management OR “organisational support” OR “work environment” OR empowerment)	Document type: Article; no publication‐date restriction. English‐language eligibility applied during screening.	15 January 2026	31 March 2026	603
Web of Science	TS=(nurse∗ OR “registered nurse” OR “clinical nurse” OR “staff nurse∗”) AND TS=(“career success” OR “career progression” OR “career advancement” OR “career development”) AND TS=(leadership OR management OR “organisational support” OR “work environment” OR empowerment)	Web of Science Core Collection; no publication‐date restriction. English‐language and full‐article eligibility applied during screening.	15 January 2026	31 March 2026	335

*Note:* Total database records: 2486. Additional records identified through backward citation searching (31 March 2026). Reference lists of included studies and relevant review articles were examined. Forward citation searching was not undertaken: 18. Total records identified before duplicate removal: 2504.

Backward citation searching was undertaken by examining the reference lists of all included studies and relevant review articles. Forward citation searching was not undertaken. The final search identified 2486 database records and 18 additional records through backward citation searching, giving 2504 records before deduplication.

### 2.4. Record Management and Study Selection

No dedicated systematic‐review or reference management platform was used. Database records were exported in RIS or CSV format and collated in Microsoft Excel for Microsoft 365. Potential duplicates were identified using combinations of DOI, title, author and publication year and were manually verified before removal. Two reviewers (EO and JO) independently screened titles and abstracts and subsequently assessed potentially eligible full‐text articles independently against the prespecified criteria. The reviewers were not formally blinded to study authors, journals or each other’s identities. Screening decisions were recorded separately before comparison. Disagreements were resolved through discussion, with NC supporting resolution when consensus was not initially reached. Inter‐rater agreement was not calculated.

### 2.5. Data Charting

A structured charting form was piloted on three studies and refined by the review team. EO extracted and charted data from all included studies. JO independently checked the completed extraction against every source article. Discrepancies were resolved through discussion, with NC consulted when required. Charted fields included author, year, country, aim, participants and setting, study design, career construct and measure, organisational, leadership, workforce, relational and individual factors, principal findings and methodological considerations. Table 4 presents the study‐level characteristics and principal methodological limitations.

**TABLE 4:** Characteristics of the studies included.

Author/year	Country	Aim	Participants/setting	Design; career construct/outcome and measure	Organisational, leadership and workforce‐related factors examined	Key findings relevant to career‐related outcomes	Principal methodological considerations/limitations
[19]	England	Examined internationally educated nurses′ experiences during their first two years of employment and residence in England.	773 internationally educated nurses working in England for less than two years.	Exploratory mixed‐methods online survey integrating quantitative items and qualitative responses; career development, progression and professional integration experiences; no objective measure of grade progression.	Recognition of previous experience, induction, line‐manager support, career‐development systems and workforce integration.	Participants identified recognition of transferable skills, induction, career‐development systems and line‐manager engagement as relevant to professional integration. Career‐development conversations and recognition practices varied across organisations.	Cross‐sectional, self‐reported and potentially affected by self‐selection. Career movement was not measured prospectively, and the findings cannot establish the effects of organisational practices.
[45]	China	Examined the relationship between work environment and career success among nurses working in assisted reproductive technology.	555 nurses with valid responses from 53 fertility centres across 26 provinces in mainland China.	Cross‐sectional survey using the Career Success Scale and Nursing Work Environment Scale; predominantly subjective career success.	Professional development, attendance at academic conferences, specialised competence, psychological care, organisational support, salary, welfare and work environment.	Career‐success scores were positively correlated with work‐environment scores. Regression analysis identified professional development, academic participation, psychological‐care competence, organisational support, salary and welfare as associated factors.	Cross‐sectional self‐report design prevents temporal or causal interpretation. The specialist assisted‐reproduction sample may limit applicability to the wider nursing workforce.
[5]	England	Compared career‐progression patterns and explored barriers and enablers among international nurses.	611,912 nurse employment records, including 115,171 international nurses; 21 international nurses participated in interviews.	Mixed methods combining retrospective administrative data from 2014 to 2021 with qualitative interviews; objective career progression through employment grades and subjective progression experiences.	Inclusive leadership, recognition of prior experience, organisational culture, peer support and progression systems.	Progression from Band 5 occurred less frequently and more slowly among international than domestic nurses. Interview participants described recognition, inclusion, representation and organisational support as relevant to their progression experiences.	Administrative data demonstrated grade‐movement patterns but could not explain all underlying mechanisms or confounding influences. The interview sample was small, and individual careers were not followed prospectively.
[16]	China	Explored relationships among self‐efficacy, information literacy, social support and career success.	3011 clinical nurses from 30 hospitals in northwestern China.	Cross‐sectional online survey using regression and structural equation modelling; scale‐based subjective career success.	Self‐efficacy, information literacy and social support.	Self‐efficacy and social support were positively associated with career‐success scores within the regression and structural models.	Simultaneously collected self‐reported variables create risks of common‐method bias and reverse direction. Structural equation modelling does not establish temporal or causal relationships.
[34]	China	Investigated emotional‐labour patterns and their relationship with nurses′ sense of career success.	385 community nurses.	Cross‐sectional survey with latent‐class analysis; perceived or subjective career success.	Emotional‐labour patterns.	Different emotional‐labour profiles were associated with different levels of perceived career success. Nurses in more actively engaged profiles reported higher career‐success scores.	Cross‐sectional self‐report data cannot determine direction. The modest community‐nurse sample and sample‐dependent latent classes limit generalisability.
[18]	United Kingdom	Investigated whether Black, Asian and minority ethnic nurses and midwives experienced career delay and progression barriers.	538 nurses and midwives across four U.K. hospitals.	Cross‐sectional survey; self‐reported career delay, access to progression and perceived organisational barriers.	Access to professional training, managerial support, recruitment practices and progression opportunities.	Respondents from minority ethnic groups reported career delay and barriers involving development opportunities, recruitment and managerial support.	Self‐reported cross‐sectional findings cannot confirm the timing or causes of career delay. The four‐hospital sample and inclusion of nurses and midwives limit wider transferability.
[15]	Turkey	Examined antecedents and outcomes of nurses′ subjective career success.	604 nurses from four hospitals.	Cross‐sectional path analysis; subjective career success as the principal outcome, with human capital and objective career success modelled as antecedents.	Staffing and resource adequacy, participation in hospital affairs, nurse–physician relationships, human capital and objective career success.	Practice‐environment characteristics, human‐capital indicators and objective career‐success variables were associated with subjective career success in the proposed path model.	Single‐time‐point self‐report data cannot establish temporal ordering. Relationships estimated through path analysis should not be interpreted as causal pathways.
[29]	Iran	Developed and evaluated a nursing‐specific career‐success instrument.	530 nurses employed in university hospitals.	Instrument‐development and psychometric‐validation study; multidimensional nursing career success rather than observed progression.	Scale domains included career development, quality care, self‐regulation and person‐organisation fit; these were construct dimensions rather than tested organisational exposures.	Psychometric findings supported a nursing‐specific conceptualisation extending beyond promotion to professional practice, development, self‐regulation and organisational alignment.	The study provided measurement rather than explanatory or intervention evidence. Further validation is required across cultures, healthcare systems and nursing populations.
[26]	Saudi Arabia	Examined relationships among transformational leadership, career adaptability, job embeddedness and perceived career success.	469 nurses employed in government hospitals.	Cross‐sectional survey using structural equation modelling; perceived career success.	Transformational leadership, career adaptability and job embeddedness.	The statistical model reported direct and indirect associations among transformational leadership, adaptability, embeddedness and perceived career success.	Cross‐sectional self‐report data cannot confirm the temporal sequence implied by mediation analysis. Government‐hospital findings may not transfer to other sectors or healthcare systems.
[33]	China	Examined work environment and career success among highly educated nurses.	1223 nurses with master’s or doctoral qualifications.	Cross‐sectional survey with hierarchical regression; scale‐based career success.	Work environment, academic engagement, teaching, staffing and participation in hospital affairs.	Work‐environment characteristics and academic participation were associated with career‐success scores after statistical adjustment.	The selected postgraduate sample limits applicability to the broader nursing workforce. Cross‐sectional self‐report data cannot demonstrate that environmental conditions produced career success.
[30]	Iran	Explored nurses′ understandings and experiences of career success.	27 nurses across 14 hospitals in northern Iran.	Qualitative study using content analysis; subjective and nursing‐specific meanings of career success.	Clinical competence, quality care, professional values, teamwork, development, organisationally supported progression, self‐regulation, fulfilment and work–life balance.	Participants conceptualised career success as multidimensional, encompassing clinical contribution, professional competence, organisational alignment, development, usefulness, fulfilment and work–life integration.	The small purposive and context‐specific sample was appropriate for conceptual exploration but cannot estimate prevalence or establish the relative importance of identified dimensions.
[14]	China	Examined relationships among innovative behaviour, self‐efficacy, colleague solidarity and career success.	848 nurses.	Cross‐sectional survey using structural equation modelling; scale‐based career success.	Innovative behaviour, self‐efficacy and colleague solidarity.	The structural model reported positive associations linking innovative behaviour, self‐efficacy, colleague solidarity and career‐success scores.	Cross‐sectional self‐report data are vulnerable to common‐method bias, reverse direction and unmeasured confounding. Modelled pathways do not establish causal mediation.
[13]	China	Examined relationships among structural empowerment, innovative behaviour, self‐efficacy and career success.	460 registered nurses.	Cross‐sectional survey using structural equation modelling; scale‐based career success.	Structural empowerment, innovative behaviour and self‐efficacy.	Structural empowerment was directly and indirectly associated with career‐success scores through innovative behaviour and self‐efficacy in the proposed model.	The single‐time‐point design cannot establish whether empowerment preceded career success. Reliance on self‐reported constructs increases common‐method bias.
[11]	China	Examined perceived organisational support and intention to remain, including the roles of career success and self‐esteem.	610 nurses.	Cross‐sectional survey using structural equation modelling; career success as a mediating construct and intention to remain as a workforce outcome.	Perceived organisational support, career success, self‐esteem and intention to remain.	The model reported indirect associations between perceived organisational support and intention to remain through career success and self‐esteem.	Intention to remain is not equivalent to actual retention or career progression. Cross‐sectional mediation does not demonstrate temporal or causal mechanisms.
[28]	Iran	Identified capabilities and conditions perceived as necessary for nursing career advancement.	18 nurses employed in teaching hospitals.	Qualitative study using conventional content analysis; perceived career advancement rather than observed promotion or grade movement.	Interpersonal capability, mentorship, experiential learning, clinical expertise, leadership capability, professional values and responsiveness to organisational expectations.	Participants identified clinical mastery, interpersonal effectiveness, mentoring, leadership capability and professional commitment as relevant to career advancement.	The small purposive sample captured perceptions within a particular healthcare context. The study did not observe actual career transitions or test whether the identified capabilities led to advancement.
[27]	China	Examined relationships among perceived organisational support, career success, self‐esteem and intention to remain among male nurses.	405 male registered nurses.	Cross‐sectional survey using structural equation modelling; career success as a mediating construct and intention to remain as a workforce outcome.	Perceived organisational support, career success, self‐esteem and intention to remain.	The model reported indirect associations between organisational support and intention to remain through career success and self‐esteem.	Findings from male nurses in China may not transfer to other nursing populations. Self‐reported cross‐sectional mediation cannot establish causality, and intention does not demonstrate actual retention.
[31]	United Kingdom	Explored internationally recruited neonatal nurses′ perceptions and work experiences.	13 internationally recruited neonatal nurses.	Qualitative exploratory study; professional‐development, skill‐utilisation and progression‐related experiences without a formal career‐success measure.	Professional‐development opportunities, staffing pressures and use of prior skills.	Participants valued development opportunities but described frustration when previous knowledge and expertise were underused.	The small, specialist and internationally recruited sample limits transferability. Findings represent participant experiences rather than observed effects on career progression.
[32]	United Kingdom	Explored professional and personal transformation following nurse migration.	25 Jordanian nurses who had migrated to the United Kingdom.	Qualitative interview study; professional transition and career‐related transformation rather than a standardised progression or success outcome.	Organisational adaptation, sociocultural integration, work patterns and professional identity.	Participants described professional transition as intertwined with organisational expectations, workplace adaptation and sociocultural integration.	The specific migrant group and qualitative sample limit transferability. Retrospective accounts cannot determine whether organisational conditions produced longer‐term progression.
[17]	United Kingdom	Investigated the organisational management of migrant nurses′ skills and experience.	21 Nepali migrant nurses.	Multisited ethnographic study; career progression, professional recognition, deskilling and career satisfaction.	Role allocation, recognition of previous experience, deskilling and organisational career support.	The study documented discrepancies between participants′ previous expertise and allocated roles, alongside experiences of deskilling and constrained progression.	The focused ethnographic sample provides contextual depth but does not establish prevalence or represent all migrant nurses. Findings remain closely tied to the U.K. migration and employment context studied.
[12]	China	Developed and evaluated the Chinese version of the Career Success Scale in Nursing.	1148 nurses.	Psychometric scale‐validation study; subjective and multidimensional career success, including career satisfaction and perceived organisational competitiveness.	Scale domains rather than tested organisational exposures: career satisfaction, perceived within‐organisation competitiveness and perceived external‐organisation competitiveness.	The study provided psychometric foundations for measuring nursing career success but did not examine career movement, temporal change or organisational effects.	Cross‐sectional psychometric evidence from China requires further validation across countries and healthcare systems. The study contributes measurement evidence rather than explanatory evidence about progression.

*Note:* Career progression refers to movement or development over time, including promotion or grade movement; career advancement is treated as an upward or expanded form of progression. Objective career success concerns observable attainment, while subjective career success concerns personal evaluations of achievement, recognition or satisfaction. Professional development and intention to remain are not treated as evidence of progression unless explicitly connected to a defined career outcome. The methodological considerations presented are descriptive and do not constitute a formal critical‐appraisal or quality‐rating exercise. The study [12] was retained because no publication‐date restriction was applied, the study met all eligibility criteria, and it supplied foundational psychometric evidence for a nursing‐specific career‐success measure.

### 2.6. Collation, Coding and Synthesis

Analysis combined a descriptive numerical summary with a convergent descriptive thematic approach appropriate to a scoping review, informed by guidance on scoping‐review collation and thematic synthesis [24, 25]. Coding was primarily inductive at the descriptive level, while remaining informed by the review questions and the predefined distinctions between career‐related outcomes. The unit of coding was a study‐level finding statement relevant to a defined career‐related outcome. EO entered these statements into a codebook and thematic matrix in Microsoft Excel and assigned preliminary codes. JO independently checked every code assignment and its linkage to the reported outcome. NC reviewed the developing higher‐order categories. Differences concerning code application, category boundaries or interpretation were discussed until consensus was reached. No specialist qualitative data‐analysis software was used.

Quantitative results were translated into narrative statements that preserved the outcome examined, the direction of the reported relationship and the study design. Qualitative findings were entered as the author reported concepts or themes. Both forms of evidence were compared within the same matrix. Similar codes were grouped into descriptive categories and subsequently developed into higher‐order themes. The themes were reviewed against the complete matrix to ensure that divergent findings, study context and outcome type were retained. The analysis did not calculate pooled effects, rank themes by strength or interpret the frequency of a theme as evidence of importance.

A final interpretive check distinguished three outcome categories: career progression, with career advancement treated as its upward or expanded form; objective career success; and subjective career success. Professional development, professional identity, belonging and intention to remain were mapped as related processes or outcomes unless an included study explicitly incorporated them within its career‐success construct. Career satisfaction and fulfilment were treated as subjective evaluations and as dimensions of subjective career success when this reflected the original study. Organisational support, leadership and workforce conditions were treated as reported exposures or contextual conditions rather than as measures of career progression or career success. This interpretive check informed the outcome distinctions and condition domains represented in the integrated conceptual model presented in Section 3.5.

## 3. Results

### 3.1. Study Selection

Database searching identified 2486 records, and backward citation searching identified 18 additional records. After 742 duplicates were removed, 1762 titles and abstracts were screened. Ninety six full‐text articles were assessed, of which 76 were excluded: failure to meet other inclusion criteria (*n* = 40); review articles, editorials, commentaries or opinion papers (*n* = 20); and incorrect population, outcome or focus (*n* = 16). Twenty studies were included in the review (Figure 1).

**FIGURE 1:**
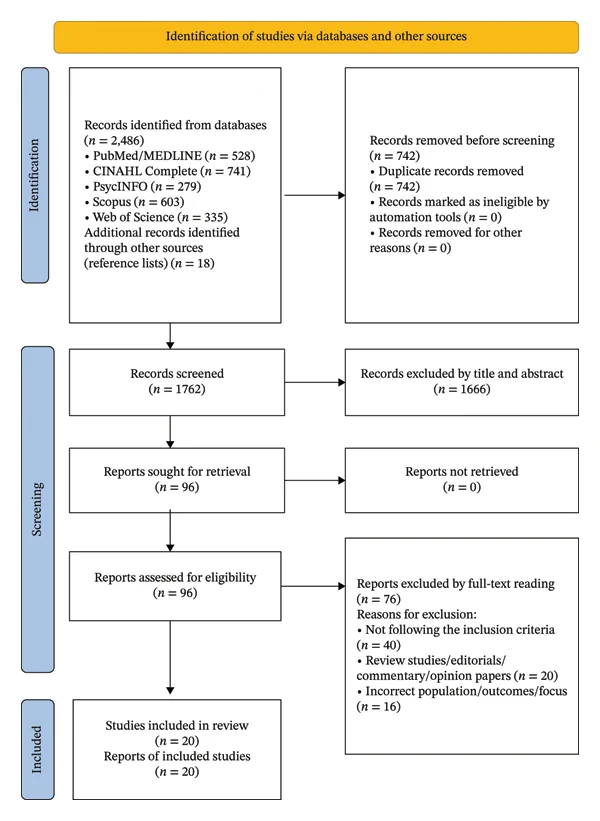
PRISMA flow diagram.

### 3.2. Characteristics and Methodological Profile

The 20 studies were published between 2014 and 2025. Thirteen used quantitative designs, comprising 11 cross‐sectional survey or modelling studies and two psychometric studies; five used qualitative designs; and two used mixed methods designs. Seven quantitative studies used structural equation modelling or path analysis [11, 13–16, 26, 27]. No intervention studies or prospective longitudinal studies were identified. Pressley et al. [5] analysed retrospective administrative data spanning several years and incorporated qualitative interviews but did not prospectively follow individual nurses′ career trajectories.

Nine studies were conducted in China, six in the United Kingdom or England, three in Iran, one in Turkey and one in Saudi Arabia. Sample sizes ranged from 13 participants in a qualitative study to 611,912 records in the quantitative component of a mixed‐methods study. Five studies focused wholly or partly on internationally recruited or migrant nurses, and one examined career delay among Black, Asian and minority ethnic nurses and midwives. Detailed study characteristics and study‐level methodological considerations are presented in Table 4.

### 3.3. Conceptualisation and Measurement of Career‐Related Outcomes

The studies included did not examine a single homogeneous outcome. Career progression was operationalised as movement or development over time within or across nursing roles, including grade movement, promotion, expanded responsibility, movement through a career pathway and recognition of prior experience. Career advancement represented the more specific upward or expanded form of this movement and was therefore treated as one possible expression of progression rather than as a synonym for professional development. These operationalisations were most evident in studies examining employment grades, delayed promotion, expanded responsibility and access to advancement opportunities [17–19, 28].

Objective career success was represented by observable attainment, including position, promotion, grade, income, professional title, administrative responsibility and external recognition. It was sometimes treated as an antecedent of subjective career success rather than as the principal outcome [15]. Promotion and grade could therefore indicate career progression when assessed as movement over time or objective career success when recorded as an attained position at a particular point. Other quantitative studies combined observable and evaluative dimensions within a total career‐success score, which reduced comparability across studies.

Subjective career success was the most common outcome in the quantitative literature. Included studies used general or nursing‐specific measures encompassing career satisfaction, perceived achievement, recognition, influence, quality work, person‐organisation fit and perceived competitiveness [12, 15, 26, 29]. Qualitative studies extended the construct to include meaningful contribution, clinical competence, usefulness, professional identity, work–life integration and fulfilment [28, 30]. Career satisfaction and fulfilment were therefore treated as subjective evaluations that could contribute to subjective career success, not as evidence that career progression had occurred. These dimensions were not defined consistently across settings.

The study [12] was retained as measurement evidence because it met all eligibility criteria and provided early psychometric evidence for a nursing‐specific career‐success scale. It was not interpreted as evidence of change, progression over time or causal relationships.

Professional development, specialist competence, academic participation, self‐efficacy, professional identity and intention to remain were variously positioned as antecedents, components, mediators or consequences. They were mapped according to the role assigned to them in each study rather than recoded as synonyms for progression or success. Similarly, organisational support, leadership and workforce environment were treated as conditions reported in relation to defined career‐related outcomes, rather than as career outcomes themselves. These distinctions form the outcome component of the integrated conceptual model presented in Figure 2 (Section 3.5).

**FIGURE 2:**
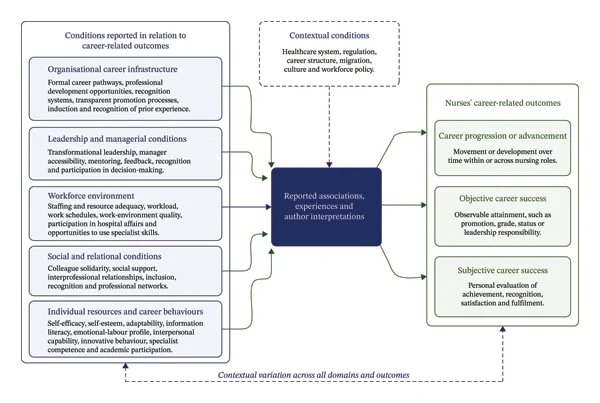
Context‐sensitive conceptual model of conditions reported in relation to nurses′ career progression and career success.

### 3.4. Reported Conditions Related to Career Outcomes

Five descriptive themes were developed from the thematic matrix. The themes present organisational, leadership, workforce, relational and individual conditions reported in relation to defined career‐related outcomes. They do not treat these conditions as alternative definitions of career progression or career success. The themes represent reported associations, participant experiences and author interpretations. Their order and frequency do not indicate effect strength, causal priority or certainty. Table 5 presents an illustration of how the themes were derived.

**TABLE 5:** Illustrative audit trail from study‐level finding statements to higher‐order descriptive themes.

Illustrative study‐level finding statements entered into the matrix	Preliminary descriptive codes	Descriptive categories	Higher‐order descriptive theme	Career outcome classification retained
Quantitative evidence: Cross‐sectional studies reported associations between structural empowerment or perceived organisational support and career‐success scores [14, 12, 11].	Organisational support; structural empowerment; access to opportunity; recognition of prior expertise; career conversations; promotion processes; development access; skill underutilisation; deskilling	Organisational support and opportunity; formal career and development systems; recognition and deployment of expertise	Organisational career infrastructure	Career progression/advancement and subjective career success. Intention to remain was retained as a related workforce outcome where reported.
Qualitative/mixed‐methods evidence: Qualitative and mixed‐methods studies reported under‐recognition of previous expertise, deskilling, inconsistent career conversations and restricted access to training or advancement [17, 31, 18, 19]				

Quantitative evidence: A cross‐sectional model reported relationships among transformational leadership, career adaptability, job embeddedness and perceived career success [26]	Transformational leadership; manager accessibility; mentoring; feedback; managerial recognition; induction support; career conversations; participation and voice	Leadership approach; manager‐mediated career support; developmental relationships; recognition and involvement	Leadership and managerial conditions	Subjective career success and career progression/advancement
Qualitative/mixed‐methods evidence: Qualitative and mixed‐methods findings described managerial recognition, mentoring, induction support, feedback and career conversations [19, 28]				

Quantitative evidence: Cross‐sectional studies reported associations involving staffing and resource adequacy, participation in hospital affairs, work‐environment quality, academic participation and specialist competence [15, 33, 45]	Staffing adequacy; resource adequacy; workload; work schedules; organisational participation; practice environment; academic opportunity; specialist‐skill utilisation; restrictive role allocation	Staffing and work design; participation and autonomy; practice and academic opportunity; utilisation of professional capability	Workforce environment	Subjective career success and career progression/advancement
Qualitative/mixed‐methods evidence: Internationally recruited nurses described staffing pressures and role allocation that restricted the use of previous expertise [31].				

Quantitative evidence: Quantitative studies reported associations between social support, colleague solidarity or interprofessional relationships and career‐success scores [14, 15, 16]	Social support; colleague solidarity; interprofessional relationships; inclusion; teamwork; interpersonal recognition; professional networks; organisational integration	Peer and interprofessional support; inclusion and integration; interpersonal recognition; professional relationships and networks	Social and relational conditions	Subjective career success and career progression/advancement. Professional identity and belonging were retained as related constructs.
Qualitative/mixed‐methods evidence: Qualitative findings linked inclusion, teamwork, professional recognition and organisational integration with career experience and professional contribution [5, 30, 32]				

Quantitative evidence: Statistical models examined self‐efficacy, self‐esteem, adaptability, information literacy, emotional labour, innovative behaviour, specialist competence and academic participation in relation to career‐success outcomes [26, 14, 34, 45, 12, 11, 33, 16]	Self‐efficacy; self‐esteem; career adaptability; information literacy; emotional labour; innovative behaviour; clinical competence; leadership capability; interpersonal capability; academic participation; professional values	Psychological resources; adaptive and proactive career behaviours; professional capability; academic and clinical engagement	Individual resources and career behaviours	Subjective career success and career progression/advancement
Qualitative/mixed‐methods evidence: Qualitative findings described clinical expertise, leadership capability, interpersonal effectiveness and professional values in accounts of advancement [28]				

*Note:* Quantitative findings were converted into narrative statements preserving the outcome, reported direction and study design. Qualitative findings were entered as author‐reported concepts or themes. Study design, country, population, outcome classification and contextual qualifications remained attached to each finding in the thematic matrix. The examples are illustrative and do not represent frequency, evidential strength or thematic importance. The wording of the preliminary codes should be checked against the saved Excel codebook before submission.

#### 3.4.1. Organisational Career Infrastructure

This theme comprised formal career pathways, access to continuing professional development, structural empowerment, recognition systems, transparent promotion processes, induction and recognition of prior experience. Quantitative studies reported associations between perceived organisational support or structural empowerment and career‐success scores [11, 13, 27]. Studies of internationally recruited nurses described inconsistent recognition of prior expertise, limited career conversations, deskilling and allocation to roles below their previous capability [17–19, 31] and reported perceived career delay and reduced access to training among racially and ethnically minoritised nurses and midwives. These findings identify organisational access and recognition as recurring conditions but do not demonstrate that a particular form of career infrastructure produces progression.

#### 3.4.2. Leadership and Managerial Conditions

Transformational leadership, manager accessibility, mentoring, feedback, recognition and participation in decision‐making formed the second theme. Pollock et al. [26] reported a cross‐sectional serial mediation model linking transformational leadership, career adaptability, job embeddedness and perceived career success. Qualitative and mixed‐methods studies described line‐manager involvement in induction, recognition of transferable experience, access to professional development and career conversations [19, 28]. Because leadership and career outcomes were measured principally through individual perceptions and at the same time point, the direction and independence of these relationships remain uncertain.

#### 3.4.3. Workforce Environment

Staffing and resource adequacy, workload, work schedules, participation in hospital affairs, work‐environment quality and opportunities to use specialist skills were reported in relation to subjective career success or career progression. In Turkey, Sönmez et al. 2021 reported associations between subjective career success and participation in hospital affairs, staffing and resource adequacy and nurse–physician relationships within a cross‐sectional path model. Chinese studies similarly reported associations involving work environment, academic participation, specialist competence and career‐success scores [33]. Internationally recruited nurses described frustration when staffing pressures or role allocation limited the use of their prior expertise [31]. These findings were based principally on individual perceptions of the work environment rather than independently measured organisational conditions.

#### 3.4.4. Social and Relational Conditions

Colleague solidarity, social support, interprofessional relationships, inclusion, recognition and professional networks comprised the fourth theme. Cross‐sectional models reported positive associations between social support or colleague solidarity and career‐success scores [14, 16]. Qualitative studies described inclusion, teamwork and recognition in relation to professional identity, meaningful contribution and access to opportunity [30, 32]. In studies of migrant nurses, relational support could not be separated fully from organisational integration and wider sociocultural adaptation.

#### 3.4.5. Individual Resources and Career Behaviours

Self‐efficacy, self‐esteem, adaptability, information literacy, emotional‐labour profile, interpersonal capability, innovative behaviour, specialist competence and academic participation were reported in relation to career‐success outcomes [13, 14, 16, 26, 28, 33, 34]. These studies described clinical expertise, leadership capability, interpersonal effectiveness and commitment to professional values as capabilities perceived to support advancement. Individual resources and behaviours were therefore frequently represented in the literature. These findings do not demonstrate that individual effort can overcome restricted opportunity structures, discrimination, nonrecognition of expertise or adverse workforce conditions.

### 3.5. Contextual Heterogeneity

The meaning and reported indicators of career progression and career success varied across national and healthcare system contexts. Chinese studies predominantly used standardised career‐success measures and cross‐sectional statistical models in hospital or clinical samples. They examined professional title, perceived organisational support, structural empowerment, work environment, social support, self‐efficacy, innovative behaviour, information literacy and emotional labour in relation to career‐success scores [11–14, 16, 27, 33, 34]. Iranian studies used qualitative and psychometric approaches that incorporated clinical contribution, professional values, self‐regulation, person‐organisation fit, meaningful work and fulfilment within career success or advancement [28–30]. The Turkish study used a cross‐sectional path model to examine subjective career success in relation to human‐capital indicators, objective career success and practice‐environment characteristics [15]. The Saudi Arabian study tested a cross‐sectional model involving transformational leadership, career adaptability, job embeddedness and perceived career success [26].

Studies conducted in the United Kingdom and England focused more frequently on migration, ethnicity, recognition of overseas qualifications and experience, access to professional development and movement through formal employment grades [5, 17–19, 31, 32], for example, examined progression through National Health Service employment grades, whereas Johnson et al. [18] reported perceived career delay and restricted access to training among Black, Asian and minority ethnic nurses and midwives. Studies of internationally recruited nurses further described deskilling, underrecognition of previous expertise, organisational integration and variable access to career‐development opportunities [17, 19, 31, 32].

These contextual differences indicate that constructs, measures and reported relationships identified in one healthcare system cannot be assumed to have equivalent meanings or operate similarly elsewhere. No included study directly tested the measurement equivalence of career‐success instruments across national healthcare systems. Figure 2 integrates the recurring condition domains and career‐related outcome distinctions identified across the included studies while retaining healthcare‐system, regulatory, career‐structure, migration, cultural and workforce policy contexts as cross‐cutting considerations. The model provides a conceptual organisation of the mapped evidence rather than a universal explanatory pathway or causal account of nurses′ career progression and success.

### 3.6. Robustness and Gaps in the Mapped Evidence

The mapped evidence had limited capacity to support explanatory or causal conclusions. Eleven quantitative studies used cross‐sectional survey or modelling designs, and most relied on self‐reported exposures and outcomes collected at a single time point [11, 13–16, 18, 26, 27, 33, 34]. These design features increased susceptibility to common‐method bias, reverse direction, selection effects and unmeasured confounding. Seven studies used structural equation modelling or path analysis to test theoretically specified relationships [11, 13–16, 26, 27]. Although these analyses examined the fit of proposed relationships, their cross‐sectional designs did not establish temporal ordering or causal effects. The two psychometric studies contributed evidence concerning the conceptual structure and measurement of nursing career success but did not examine career change, temporal progression or intervention effects [12, 29].

Five qualitative studies provided detailed accounts of career meaning, professional identity, recognition, organisational experience and perceived advancement [17, 28, 30–32]. However, these studies generally used small purposive samples within specific professional, organisational or migrant contexts. Their findings contributed contextual depth but were not intended to estimate prevalence or establish the relative importance of different career conditions. The two mixed‐methods studies also extended the breadth of the evidence but had important limitations. Pressley et al. [5] combined retrospective administrative data on employment‐grade progression with interviews, whereas Kristoffersen [19] used a cross‐sectional online survey containing quantitative items and open‐text responses. Neither study prospectively followed individual nurses through defined career transitions or evaluated the effects of an organisational intervention.

No included study evaluated whether structured career pathways, mentorship, leadership development or changes to workforce conditions improved career progression or career success. No prospective longitudinal study was identified. As shown in Table 3, the evidence was concentrated in China, the United Kingdom or England, Iran, Turkey and Saudi Arabia. No included study was conducted in the Americas, Africa or Oceania and most European healthcare systems were not represented. These geographical and methodological gaps limit the transferability of the mapped findings and reinforce the need to interpret them as reported associations and experiences rather than as established explanatory relationships.

## 4. Discussion

This review mapped organisational career infrastructure, leadership and managerial conditions, workforce environment, social and relational conditions and individual resources and career behaviours in relation to nurses′ career‐related outcomes. The integrated conceptual model in Figure 2 positions these domains alongside three distinct outcomes: career progression or advancement, objective career success and subjective career success. Healthcare system, regulatory, career structure, migration, cultural and workforce policy contexts extend across the model because they shape how career opportunities and outcomes are defined and interpreted. The model represents reported associations, experiences and author interpretations rather than a hierarchy of determinants, causal sequence or universal pathway through which progression occurs.

The distinction between career progression and career success has important implications for interpreting the literature. Wider career scholarship differentiates observable attainment from personal evaluations of achievement and satisfaction [7, 8, 35]. Findings extend subjective success to clinical contribution, professional values, meaningful work and fulfilment [12, 29, 30]. This broader interpretation reflects the clinical and relational character of nursing practice. Nevertheless, combining progression, satisfaction, identity and professional development can obscure whether a study concerns career movement, readiness for movement or personal evaluation.

Recurring reports concerning organisational career infrastructure are consistent with Kanter’s [36] account of access to opportunities, information, support and resources. Reviews of continuing professional development also indicate that learning is more likely to influence practice when nurses have relevant opportunities and scope to apply their learning [3, 37]. Recognition of prior experience, transparent promotion processes and career conversations were similarly reported alongside career‐related outcomes. Yet most included studies measured perceptions at a single time point. Organisational career infrastructure remains an important area for investigation, but the evidence does not establish that a particular pathway or development programme produces progression.

Leadership and workforce conditions showed a similar pattern. Evidence associates relational, authentic and transformational leadership with job satisfaction and work‐environment outcomes [38–40]. Such outcomes remain relevant to employment experience but are not equivalent to progression. Included studies reported managerial recognition, mentoring, career conversations, participation and staffing adequacy in relation to subjective success or perceived advancement [15, 19, 26]. Cross‐sectional self‐report data cannot determine direction. Supportive environments may contribute to positive career evaluations, while nurses who feel successful may evaluate their work environment more favourably. Colleague solidarity, social support and interprofessional relationships were also reported in relation to career‐success scores and career experiences [14–16], although these relational conditions were predominantly examined through cross‐sectional self‐report.

Individual resources such as self‐efficacy, adaptability and innovative behaviour featured prominently. Social cognitive and career construction perspectives recognise that agency and adaptability can support career action [41]. However, evidence concerning migrant and minoritised nurses shows why individualised explanations are insufficient. Deskilling, delayed advancement and underrecognition of overseas expertise were reported despite prior capability [5, 17, 18]. Wider reviews similarly identify recognition, regulation, organisational integration and workplace support as important contextual conditions [5, 42, 43]. Career agency should be interpreted in relation to the opportunities and constraints within which nurses work.

Contextual heterogeneity further limits generalisation. Professional grades, educational routes, promotion systems, migration arrangements and cultural understandings of success differ across healthcare systems. Cross‐cultural career research supports multidimensional accounts of subjective success and culturally appropriate measurement [44–46]. No included study tested measurement equivalence, while evidence from five national settings restricts transferability. The predominance of cross‐sectional studies, self‐reported outcomes and single‐time‐point modelling also limits temporal and causal interpretation. Longitudinal research should examine grade, role and responsibility changes alongside subjective success, while intervention and implementation studies should evaluate organisational initiatives across workforce groups and healthcare contexts.

## 5. Implications for Nursing Management

The findings identify areas that nursing managers can examine within local workforce systems, rather than interventions with established effectiveness. Organisations can review whether career pathways and promotion criteria are transparent, whether development and mentoring opportunities are accessible across employment grades, and whether career conversations form part of routine management practice. Recognition and appropriate use of previous expertise require particular attention for internationally educated nurses. Workforce reviews should also examine whether staffing pressures, restricted role utilisation or limited protected time prevent nurses from accessing development opportunities.

Career initiatives should be linked to clearly defined outcomes before implementation. Participation in professional development should be distinguished from movement into a new grade, role or level of responsibility. Evaluation should include both objective indicators, such as promotion or expanded responsibility, and subjective evaluations of career success. Outcomes should be examined over time and, where possible, disaggregated by ethnicity, country of qualification, employment grade, specialty and other relevant workforce characteristics. This approach would help identify unequal access, differential outcomes and unintended consequences.

Current evidence does not demonstrate that structured pathways, mentoring, leadership development or workforce changes improve progression, retention, workforce sustainability or leadership succession. Nursing managers should consequently introduce such initiatives with prospective evaluation rather than assume benefit. Mixed‐methods implementation studies could establish who accesses an initiative, whether career movement follows, how nurses experience the process and how organisational context contributes to variation in outcomes.

### 5.1. Limitations

Some limitations of the review process should be considered. The search covered five multidisciplinary databases, but restriction to English‐language, peer‐reviewed journal articles may have excluded relevant evidence. Grey literature, dissertations and conference materials were excluded. Backward citation searching identified additional records, but forward citation searching was not undertaken. Studies using less common career terminology may consequently have been missed despite the use of broad search terms.

No formal critical appraisal or certainty of evidence assessment was undertaken. Although such appraisal is not mandatory for a scoping review, the review cannot provide study‐level quality ratings or conclusions about certainty. Methodological characteristics and principal limitations were charted to support appropriately cautious interpretation.

The conceptual diversity of the literature required the review team to categorise constructs that were not always clearly defined in the primary studies. Use of a codebook, thematic matrix, independent checking and consensus discussion strengthened transparency, but interpretive judgement remained unavoidable. The a priori protocol was neither registered nor published, limiting external verification of the planned methods. The synthesis also brought together diverse populations, healthcare systems, measures and study designs. The resulting themes organise the literature but should not be interpreted as universally transferable or as evidence that the reported conditions have equivalent meanings across settings.

## 6. Conclusion

This scoping review mapped organisational career infrastructure, leadership and managerial conditions, workforce environment, social and relational conditions and individual resources and career behaviours in relation to nurses’ career‐related outcomes. The synthesis distinguishes career progression, objective career success and subjective career success, while positioning professional development, professional identity, belonging and intention to remain as related but non‐equivalent constructs. Future research should employ explicit outcome definitions, context‐sensitive measures and longitudinal or intervention designs capable of examining actual career movement across diverse nursing populations and healthcare systems.

## Author Contributions

Elizabeth Bukola: conceptualisation, methodology, investigation, formal analysis, writing–original draft. Jude Ominyi: methodology, formal analysis, data curation, supervision, writing–review and editing. Noreen Cushen‐Brewster: methodology, data curation, supervision, writing–review and editing.

## Funding

This research received no specific grant from any funding agency in the public, commercial or not for profit sectors.

## Disclosure

This scoping review was reported in accordance with the Preferred Reporting Items for Systematic Reviews and Meta‐Analyses extension for Scoping Reviews (PRISMA‐ScR) [23]. The completed PRISMA‐ScR checklist is provided as a supporting file.

## Ethics Statement

Ethics approval was not required for this scoping review because it analysed previously published literature and did not involve human participants, identifiable personal data or primary data collection.

## Conflicts of Interest

The authors declare no conflicts of interest.

## Supporting Information

Additional supporting information can be found online in the Supporting Information section.

## Supporting information


**Supporting Information** PRISMA_2020_checklist_1.

## Data Availability

All data supporting the findings of this review are presented within the article and its supporting information.
